# DNA Replication Stress Is a Determinant of Chronological Lifespan in Budding Yeast

**DOI:** 10.1371/journal.pone.0000748

**Published:** 2007-08-15

**Authors:** Martin Weinberger, Li Feng, Anita Paul, Daniel L. Smith, Robert D. Hontz, Jeffrey S. Smith, Marija Vujcic, Keshav K. Singh, Joel A. Huberman, William C. Burhans

**Affiliations:** 1 Department of Cell Stress Biology, Roswell Park Cancer Institute, Buffalo, New York, United States of America; 2 Department of Cancer Biology, Roswell Park Cancer Institute, Buffalo, New York, United States of America; 3 Department of Biochemistry and Molecular Genetics, University of Virginia Health System, Charlottesville, Virginia, United States of America; 4 Department of Cancer Genetics, Roswell Park Cancer Institute, Buffalo, New York, United States of America; University of Minnesota, United States of America

## Abstract

The chronological lifespan of eukaryotic organisms is extended by the mutational inactivation of conserved growth-signaling pathways that regulate progression into and through the cell cycle. Here we show that in the budding yeast *S. cerevisiae*, these and other lifespan-extending conditions, including caloric restriction and osmotic stress, increase the efficiency with which nutrient-depleted cells establish or maintain a cell cycle arrest in G1. Proteins required for efficient G1 arrest and longevity when nutrients are limiting include the DNA replication stress response proteins Mec1 and Rad53. Ectopic expression of *CLN3* encoding a G1 cyclin downregulated during nutrient depletion increases the frequency with which nutrient depleted cells arrest growth in S phase instead of G1. Ectopic expression of *CLN3* also shortens chronological lifespan in concert with age-dependent increases in genome instability and apoptosis. These findings indicate that replication stress is an important determinant of chronological lifespan in budding yeast. Protection from replication stress by growth-inhibitory effects of caloric restriction, osmotic and other stresses may contribute to hormesis effects on lifespan. Replication stress also likely impacts the longevity of higher eukaryotes, including humans.

## Introduction

In eukaryotic organisms as diverse as yeasts and mice, lifespan is extended by caloric restriction or mutations that inactivate conserved growth signaling pathways (reviewed in [1], [2]). Conserved elements of these pathways include RAS proteins and members of the AKT/PKB family of kinases that function in glucose signaling in yeast and insulin/insulin-like growth factor (I-(IFG-1)) signaling in higher eukaryotes, as well as elements of TOR-dependent nitrogen signaling pathways. Mutations that promote, rather than inhibit, signaling in these pathways shorten lifespan.

The mechanisms by which caloric restriction or mutational inactivation of growth signaling pathways extend lifespan remain unclear. Both induce stress responses that depend on heat shock and other proteins, including proteins that mitigate oxidative stress [3], [4], [5]. The induction of oxidative stress responses, in particular, might promote longevity by protecting against reactive oxygen damage to lipids, protein and DNA, all of which accumulate oxidative damage during aging [6]. Oxidative damage to DNA could be responsible for some of the increased genome instability associated with aging in all eukaryotes [7]. However, a definitive role for oxidative damage in aging has not yet been established [6], [8]. For example, in mice, a mutation in the superoxide-scavenging enzyme *SOD2* elevates oxidative damage to DNA, but does not shorten lifespan [9]. The absence of a correlation between levels of reactive oxygen and longevity has also been noted in *Drosophila* [10]. It also remains unclear how cellular responses to other stresses might inhibit age-dependent increases in DNA damage and promote longevity.

The budding yeast *Saccharomyces cerevisiae* has proved to be a valuable model organism for investigating conserved pathways regulating lifespan in all eukaryotes. Replicative lifespan in budding yeast is assessed by determining the number of times cells divide in the presence of nutrients before they senesce and die [11] via an apoptotic-like mechanism [12]. An important factor determining replicative lifespan is DNA replication stress–i.e., inefficient DNA replication that leads to replication fork stalling–which stimulates recombination at a replication fork barrier in the rDNA locus ([13] and references therein).

Chronological lifespan in budding yeast is distinct from replicative lifespan and is measured by assessing the viability of cells driven into a growth-arrested state by nutrient depletion [11]. The quiescent state of nutrient-depleted budding yeast cells shares features with postmitotic cells in higher eukaryotes, including growth arrest of a large fraction of cells with a G1 content of DNA. Nutrient-depleted budding yeast cells can survive in this quiescent state for an extended time, during which replenishing the medium restores growth. Similar to replicatively aged budding yeast cells, chronologically aged cells eventually die via an apoptotic-like mechanism that includes degradation of DNA [14], [15].

Most efforts to understand how alterations in nutrient-signaling pathways impact chronological lifespan in budding yeast have focused on changes in nutrient depletion-induced stress resistance, especially resistance to oxidative stress. Stress resistance in this organism is mediated in large part by the “Rim15 regulon”, which is upregulated by the Rim15 kinase in response to depletion of nutrients [16], [17]. The Rim15 regulon includes a number of genes that, like *RIM15* [5], promote longevity in the chronological aging model. This includes genes encoding the stress-responsive transcription factors Msn2 and Msn4 and genes induced by Msn2 and Msn4, such as *SOD1* and *SOD2* encoding superoxide dismutases that mitigate oxidative stress [4]. In the presence of glucose and other nutrients, Rim15 kinase activity and the induction of stress resistance are inhibited by the Ras-cAMP glucose signaling pathway, the constitutive activation of which shortens lifespan [18]. Furthermore, nuclear localization of Rim15, which is required for induction of the Rim15 regulon, is negatively regulated by Sch9 in the presence of glucose [19]. Abrogation of this negative regulatory mechanism in *sch9Δ* cells extends chronological lifespan [5]. Rim15 also is negatively regulated by the nitrogen-sensitive TOR-dependent nutrient signaling pathway [20], inhibition of which also extends chronological lifespan [21], and by Pho80-Pho85 cyclin-CDK-dependent pathways that respond to phosphate levels [22].

Chronological lifespan extension associated with the induction of oxidative stress responses by Rim15 and other proteins is consistent with the longstanding “free radical” theory of aging, which posits oxidative damage as a major determinant of lifespan in all eukaryotes [23]. However, Rim15 also mediates the G1 arrest induced by nutrient deprivation in budding yeast (reviewed in [17]). In addition to the G1 arrest induced when medium is depleted of nutrients [24], Rim15 is also required for G1 arrest when TOR-dependent nitrogen-signaling pathways are inhibited [19]. Phosphate starvation also leads to the activation of Rim15 and entry into a G0-like state [22]. Thus, Rim15 integrates signals from several nutrient-signaling pathways to arrest cells in the G1 phase of the cell cycle during nutrient deprivation, in addition to its induction of oxidative and other stress responses. The possibility that alterations in the Rim15-dependent G1 arrest induced by nutrient deprivation impact chronological lifespan when nutrient signaling pathways are altered has not been investigated previously.

The shorter lifespan of nutrient-depleted *rim15Δ* cells [5] is accompanied by growth arrest throughout the cell cycle, including S phase [19]. A similar growth arrest throughout the cell cycle is observed during nutrient depletion of cells harboring the constitutively activating *RAS2* ^val19^ mutation, which also shortens chronological lifespan [25]. Growth arrest of *RAS2* ^val19^, *rim15Δ* and other cells in S phase could be caused by a reduction in the levels of nucleotides and other factors required for efficient DNA synthesis, which would lead to replication stress. As in other eukaryotes, replication stress promotes genome instability [26], [27] and apoptosis [28] in budding yeast. Both genome instability and apoptosis are characteristic features of chronological aging in this organism, [14], [15], [29] as well as in many other eukaryotes [8].

Based on these considerations, we hypothesized that inhibition of nutrient-signaling pathways can extend the chronological lifespan of budding yeast by inducing a more efficient nutrient depletion-induced G1 arrest. This more efficient G1 arrest would protect against replication stress-induced genome instability and apoptosis by reducing the growth arrest of cells in S phase with incompletely replicated chromosomes. To test this hypothesis, we measured the frequency with which cells arrest in G1 and undergo apoptosis during nutrient depletion under conditions previously shown to alter the chronological lifespan of this organism, including caloric restriction and osmotic stress. We also examined the frequency with which nutrient depletion induces G1 arrest and apoptosis in strains defective in responses to DNA replication stress. The results of these experiments demonstrated that nutrient depletion causes replication stress in cells that fail to enter into or maintain a G1 arrest when deprived of nutrients and instead arrest growth in S phase. They suggest that, in addition to its role in replicative aging in budding yeast, replication stress is an important factor determining chronological lifespan in this organism.

## Results

### Correlation between lifespan and efficiency of G1 arrest in nutrient-depleted cells

*SCH9* encodes a kinase that promotes glucose and nitrogen signaling in budding yeast in parallel with the Ras-cAMP glucose-sensing pathway ([20] and references therein). Sch9 is an orthologue of AKT/PKB kinases that function in growth signaling downstream of IGF-1 in higher eukaryotes (reviewed in [1]). Deletion of *SCH9* enhances Rim15-dependent stress responses and extends chronological lifespan [5], similar to the lifespan-extending effects of some mutations in AKT homologues in higher eukaryotes [1], [2]. To test whether deletion of *SCH9* would increase the efficiency of G1 arrest during nutrient depletion (as predicted by our hypothesis), we compared the extent of this arrest in wild type and *sch9Δ* cells by determining budding indices (fraction of budded cells), since cells with buds have passed Start and have entered or proceeded through S phase. We also employed flow cytometry to determine the proportion of nutrient-depleted cells that arrested with a G1 content of DNA. Measurements of cellular DNA content also revealed cells undergoing apoptotic DNA degradation indicated by less than a G1 DNA content.

**Figure 1:**
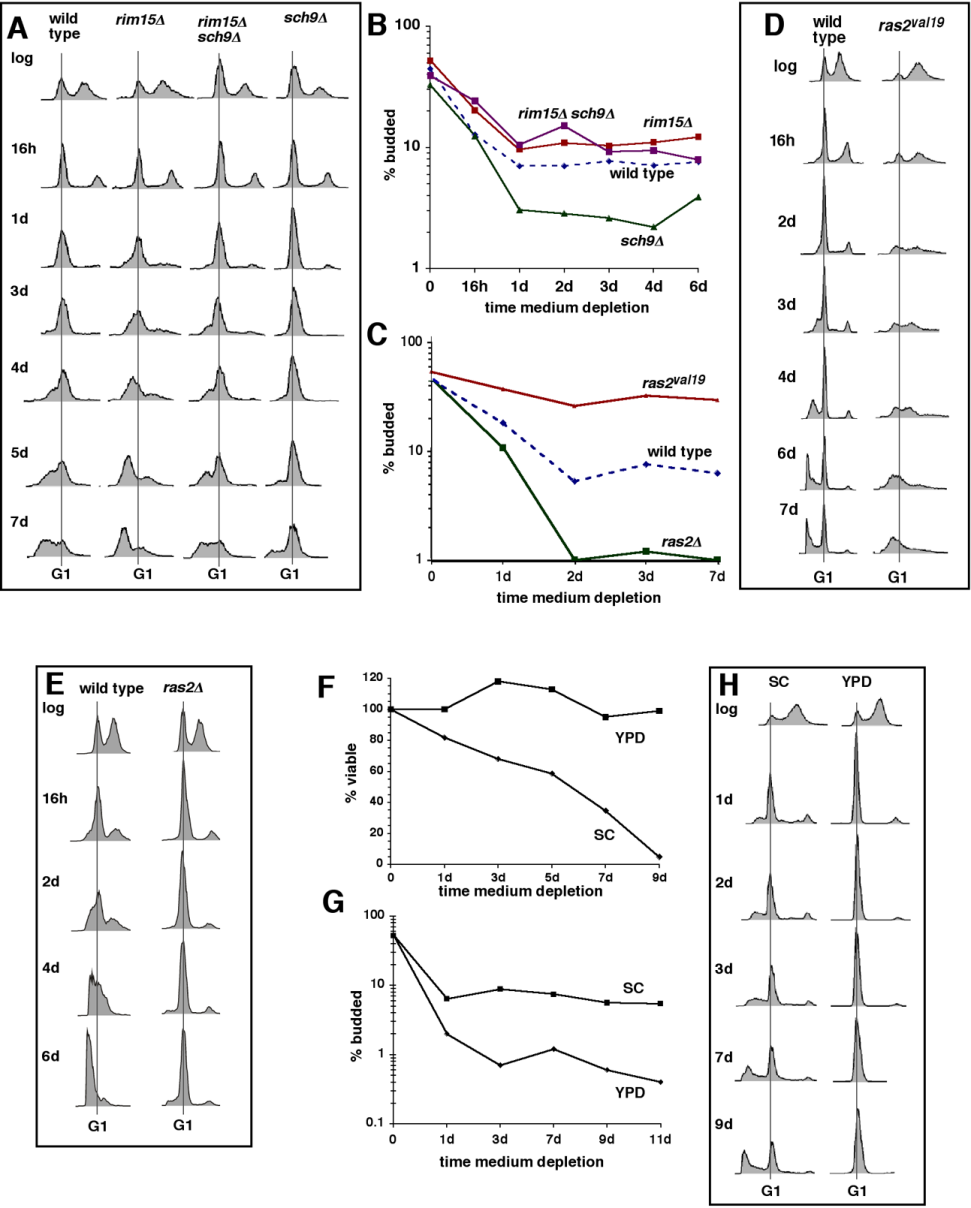
Chronological lifespan is related to the efficiency with which nutrient-depleted cells arrest in G1. A. Changes in DNA content during nutrient depletion of wild-type (DBY746) cells and cells harboring deletions of *RIM15* and/or *SCH9,* which was previously shown to shorten (*rim15Δ*) or lengthen (*sch9Δ*) chronological lifespan [5]. Length of time cells were depleted of nutrients after establishing exponential cultures (“log”) is indicated to the left. B. Budding indices of cells in Panel A. C. Budding indices of wild-type cells and cells harboring the *ras2Δ* or *RAS2* ^val19^ mutations. D. Changes in DNA content during nutrient depletion of wild-type cells and cells harboring the *RAS2* ^val19^ mutation. E. Changes in DNA content of wild type and *ras2Δ* cells during nutrient depletion. F–H. Viability (F), budding index (G) and DNA content (H) of wild-type cells nutrient depleted in synthetic complete medium (“SC”) or rich medium (“YPD”). Data in this and subsequent figures are representative of the data from three or more independent experiments unless noted otherwise.

Consistent with our hypothesis, we found that during nutrient depletion, *sch9Δ* cells arrested with a G1 content of DNA more efficiently than did wild-type cells. A more efficient G1 arrest in *sch9Δ* compared to wild type cells was indicated by the increased height and narrowness of the G1 DNA-content peak in flow cytometry profiles (Fig. 1A) in parallel with a reduced budding index (Fig. 1B). As reported earlier by Fabrizio *et al.* [5], we found that *sch9Δ* cells had a longer chronological lifespan than wild-type cells (Fig. S1). Also consistent with lifespan extension in *sch9Δ* cells, we found that at later times, *sch9Δ* cells suffered less apoptotic DNA degradation (Fig. 1A).

Rim15 is a member of the PAS kinase family containing PAS domains (reviewed in [17]). PAS domains are highly conserved regulatory modules that respond to a variety of stimuli, including oxygen, redox status and energy levels. As described above, Rim15 integrates signals from several nutrient-signaling pathways to regulate stress responses and withdrawal from the cell cycle. In the presence of nutrients, activation of Rim15 and the induction of stress responses are inhibited by Sch9 and other elements of nutrient signaling pathways. During nutrient depletion, cells deleted of *RIM15* have a shorter lifespan [5] and arrest throughout the cell cycle, which was indicated by a higher budding index compared to wild type cells [19]. We also found that nutrient-depleted *rim15Δ* cells exhibit a higher budding index (Fig. 1B). In addition, we found that compared to wild type cells, *rim15Δ* cells arrest less efficiently with a G1 content of DNA and undergo more rapid apoptotic DNA degradation (Fig. 1A). Deletion of *SCH9* from *rim15Δ* cells suppresses their shortened lifespan ([5]. Compared to *rim15Δ* cells, *sch9Δ rim15Δ* cells exhibited a more efficient G1 arrest and a reduced frequency of apoptotic DNA degradation during nutrient depletion (Figs. 1A and B).

Ras2 plays a role in a glucose-signaling pathway that functions upstream of Rim15 in parallel with the Sch9 pathway (reviewed in [30]). Similar to deletion of *SCH9*, deletion of *RAS2* extends the chronological lifespan of nutrient-depleted budding yeast cells [18]. We found that it also decreases budding index, indicating a tighter G1 arrest (Fig. 1C). A tighter G1 arrest in *ras2Δ* cells was detected by flow cytometry as well (Fig. 1E). In contrast, constitutive activation of Ras2 by the *RAS2* ^val19^ mutation shortens chronological lifespan [18]. In agreement with an earlier observation [25], we found that the *RAS2* ^val19^ mutation decreased the efficiency of G1 arrest during nutrient depletion (Fig. 1C and D).

A relationship between chronological lifespan and efficiency of G1 arrest during nutrient depletion was also suggested by the effects of another experimental manipulation previously shown to decrease the chronological lifespan of nutrient-depleted cells—culturing cells in defined (SC) medium rather than rich (YPD) medium [31], [32]. Although the basis for the decreased chronological lifespan in SC medium is not clear, nutrient-depleted cells cultured in this medium exhibit a higher metabolic rate, which correlates with shorter chronological lifespan [18]. The shorter lifespan of DBY746 cells in SC compared to YPD medium (Fig. 1F) was accompanied by a less efficient G1 arrest (Fig. 1G,H). It was also accompanied by extensive apoptotic DNA degradation, which was absent from cells cultured in YPD medium at the same time points (Fig. 1H). These effects were also detected in a second genetic background (W303; not shown).

In combination, these observations establish a strong correlation between chronological lifespan and efficiency of G1 arrest under nutrient-limiting conditions.

### Caloric restriction and osmotic stress extend chronological lifespan in parallel with a more efficient G1 arrest during nutrient depletion

**Figure 2:**
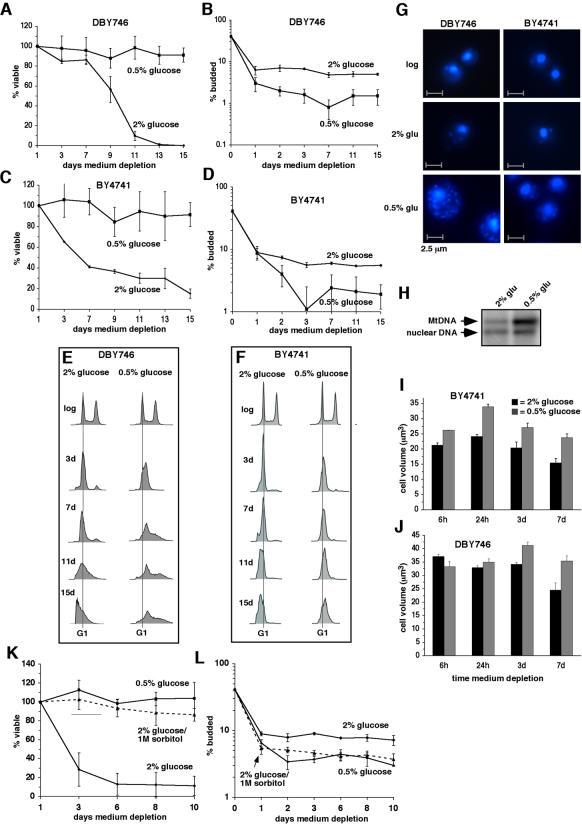
Caloric restriction and osmotic stress increase the efficiency with which nutrient-depleted cells arrest in G1. A–F. Viability (A and C), budding index (B and D) and DNA content (E and F) of DBY746 (A, B and E) and BY4741 (C, D and F) cells cultured in medium containing 2% compared to 0.5% glucose at beginning of experiment. Data in A–D are means and standard errors for three independent experiments. G. DAPI stained cells from log phase cultures cultured in medium containing 2% glucose (day 0) or after 3 days of depletion of medium containing 2% or 0.5% glucose. H. Southern blot analysis of mitochondrial and nuclear DNA content in DBY746 cells cultured for three days in medium containing 2% or 0.5% glucose. I and J. Mean volume of BY4741 (I) and DBY746 (J) cells cultured in medium containing 2% or 0.5% glucose. K and L. Effects of increased osmotic stress during nutrient depletion (produced by adding 1M D-sorbitol to medium) on viability (K) and budding index (L) of BY4741 cells.

Caloric restriction extends the replicative and chronological lifespans of budding yeast, as well as the chronological lifespans of most other eukaryotic organisms [33]. To determine whether caloric restriction also induces a more efficient G1 arrest during nutrient depletion, we assessed the effects of long-term culture of budding yeast cells in medium containing a reduced concentration of glucose (0.5% instead of 2%). Reducing the concentration of glucose to 0.5% extended the chronological lifespan of cells in the DBY746 background (Fig. 2A) at the same time that it reduced the budding index of these cells (Fig. 2B). Similar results were obtained in the BY4741 genetic background (Fig. 2C and D). FACS analysis of DNA content indicated that in both genetic backgrounds, cells cultured in 0.5% glucose more slowly underwent apoptotic DNA degradation indicated by fewer cells with less than a G1 content of DNA (Fig. 2E and F). Therefore, similar to mutational inactivation of Sch9-or Ras2-dependent nutrient signaling pathways, caloric restriction extends chronological lifespan of budding yeast in concert with a more efficient G1 arrest and less frequent apoptosis.

Unlike cells in which lifespan was extended by deletion of *SCH9* or *RAS2*, however, calorie-restricted DBY746 cells exhibited a progressive age-dependent increase in DNA content (Fig. 2E). A progressive increase in DNA content was observed in calorie-restricted BY4741 cells as well, although this increase occurred more slowly (Fig. 2F). An increase in nuclear DNA content is not consistent with the reduced budding index of calorie-restricted cells, which indicates less frequent cell division. In mammals (including humans), caloric restriction stimulates mitochondrial biogenesis [34], [35], [36]. To determine whether increased mitochondrial DNA associated with mitochondrial biogenesis might be responsible for the increased DNA content of calorie-restricted budding yeast cells, we stained cells with the DNA-specific dye DAPI. A substantial increase in the number of DAPI signals from non-nuclear, cytoplasmic DNA was apparent in nutrient-depleted DBY746 and BY4741 cells cultured for three days in medium containing 0.5% glucose compared to 2% glucose (Fig. 2G). Southern blot analysis of DNA isolated from DBY746 cells at this time point confirmed that cells cultured in medium containing 0.5% glucose contained more mitochondrial DNA relative to nuclear DNA compared to cells cultured in medium containing 2% glucose (Fig. 2H). We conclude that the increased DNA content detected by FACS under caloric restriction conditions corresponds to increased mitochondrial DNA.

Microscopic inspection of DAPI-stained cells suggested that caloric restriction also increased the size of cells in both genetic backgrounds (Fig. 2G). This was confirmed by measurements of cell volume (Fig. 2I and J). The G1 cyclin *CLN3* is downregulated during nutrient depletion, and this downregulation occurs more rapidly in calorie-restricted cells [37]. Downregulation of *CLN3* in cycling cells prolongs G1 and increases cell size [38], [39]. Therefore, the larger size of calorie-restricted cells is consistent with a tighter G1 arrest associated with more rapid downregulation of *CLN3*.

Similar to caloric restriction, increasing the osmolarity of culture medium by adding sorbitol (a nonmetabolizable sugar alcohol) extends both replicative [40] and chronological [41] lifespans of budding yeast. The effects of osmotic stress on replicative lifespan may be mechanistically related to those of caloric restriction [40]. In the absence of nutrient depletion, osmotic stress inhibits growth by inducing a G1 arrest [42], [43]. This suggested the possibility that the effects of sorbitol on chronological lifespan of nutrient-depleted cells might be related to a tighter G1 arrest. Consequently, we examined the effects of sorbitol on nutrient depletion-induced G1 arrest in parallel with effects on viability. As reported previously [41], adding sorbitol to medium extended the chronological lifespan of BY4741 cells to a similar extent compared to caloric restriction (Fig. 2K). Also similar to caloric restriction, lifespan extension by sorbitol was accompanied by a reduction in budding index (Fig. 2L) and in the number of cells undergoing apoptotic DNA degradation (Fig. S2). These findings extend the correlation between chronological lifespan extension, less frequent apoptosis and more efficient G1 arrest.

### Induction of DNA replication stress by nutrient depletion

One of the potential benefits of the G1 arrest induced by nutrient depletion is that it would protect cells from replication stress they would suffer if they were in S phase under suboptimal conditions for replicating DNA. These conditions might include the depletion from medium of substrates required for synthesis of dNTPs. Nutrient depletion also downregulates the transcription of many genes encoding proteins required for DNA replication [44], [45], [46]. These genes include *RNR1* encoding the large subunit of ribonucleotide reductase, which is required for the synthesis of dNTPs. The combined effects of reduced concentrations of substrates for DNA synthesis and downregulation of genes encoding proteins required for DNA replication is expected to produce replication stress in nutrient-depleted cells that failed to enter into or maintain a G1 arrest, and arrest growth in S phase instead.

**Figure 3:**
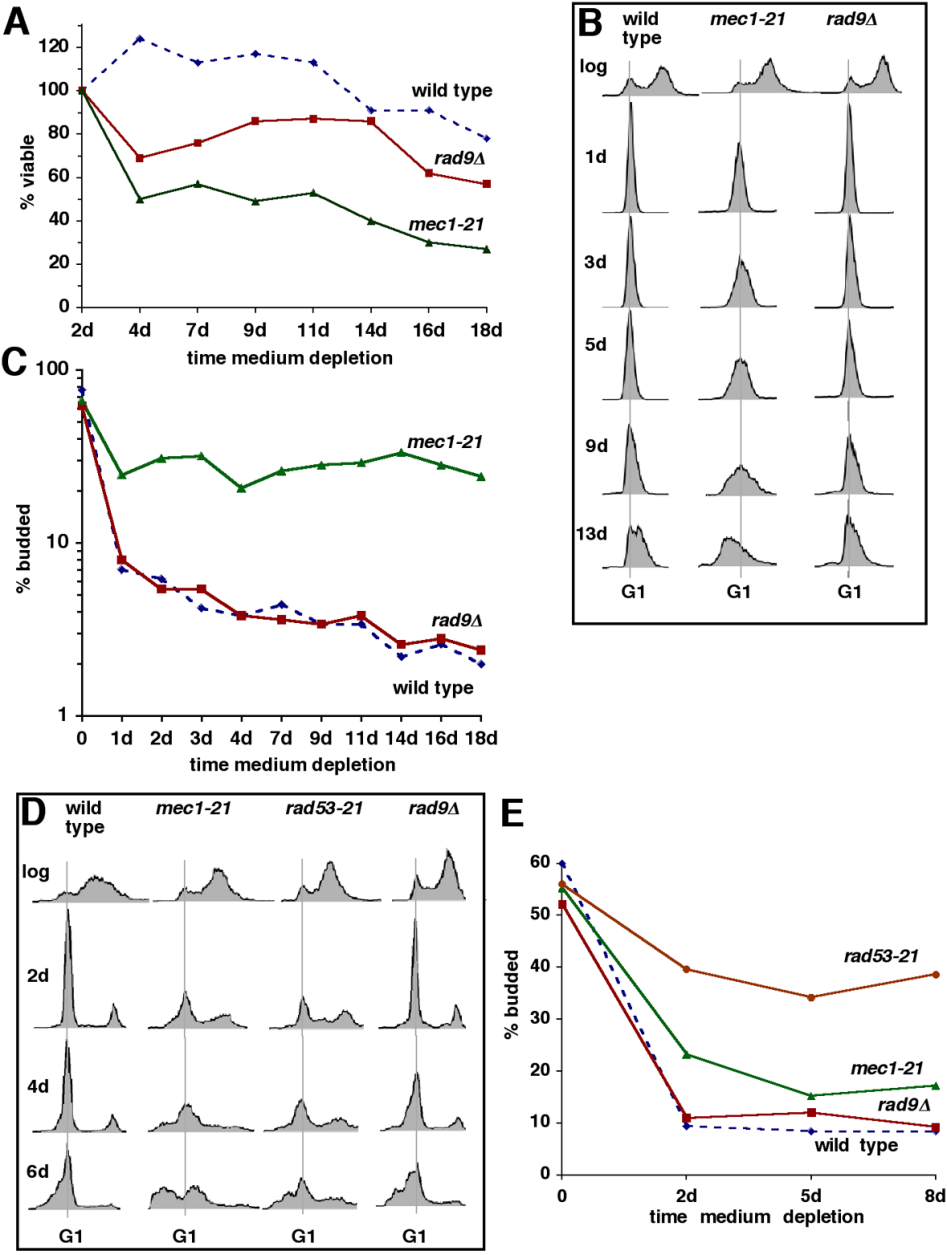
Nutrient depletion induces DNA replication stress. A–C. Viability (A), DNA content (B) and budding index (C) of wild-type (W303) cells and cells with mutations in *MEC1* or *RAD9* undergoing nutrient depletion in YPD medium. D&E. DNA content (D) and budding index (E) of wild-type, *mec1-21*, *rad53-21* and *rad9Δ* cells undergoing nutrient depletion in SC medium.

The *MEC1* gene encodes a protein required for cellular responses to both replication stress and DNA damage. *RAD9* encodes a protein required for responses to DNA damage, but not replication stress (reviewed in [47]). To determine whether nutrient depletion induces replication stress, we compared the phenotypes of nutrient-depleted wild type, *mec1-21* and *rad9Δ* cells. During nutrient depletion in YPD medium, *mec1-21* cells died more rapidly than wild-type or *rad9Δ* cells (Fig. 3A). More rapid cell death of *mec1-21* cells occurred in parallel with a less efficient arrest with a G1 content of DNA (Fig. 3B) and an elevated budding index (Fig. 3C). At later time points, *mec1-21* cells also underwent apoptotic DNA degradation, which was largely absent from wild-type or *rad9Δ* cells at the same time points (Fig. 3B). Nutrient-depleted *rad9Δ* cells exhibited budding indices similar to those of wild type cells (Fig. 3C). This is consistent with the absence of a role for Rad9 in cellular responses to replication stress, in contrast to the roles of Mec1 and Rad53. However, *rad9Δ* cells died more rapidly than wild type cells, although not as rapidly as *mec1-21* cells (Fig. 3A). This suggests that nutrient depletion induces DNA damage, in addition to replication stress.

*RAD53* encodes a protein that functions downstream of Mec1 in cellular responses to DNA damage and replication stress. [47]. Similar defects in G1 arrest were observed in *rad53-21* and *mec1-21* compared to wild-type and *rad9Δ* cells during nutrient depletion in SC medium (Fig. 3D and E). Furthermore, apoptotic DNA degradation was accelerated in *rad53-21* and *mec1-21* cells. Apoptotic DNA degradation in wild type, *mec1-21* and *rad9Δ* strains was also accelerated in cells cultured in SC compared to YPD medium. Together with the data presented in Figs. 3A–C, these findings indicate that nutrient depletion induces replication stress, which is enhanced in SC compared to YPD medium.

An essential function shared by Mec1 and Rad53 is to maintain sufficient dNTP pools to complete DNA replication during S phase [47]. Maintenance of dNTP pools requires the induction of Rnr1 activity by Mec1-and Rad53-dependent pathways that inactivate Sml1, an inhibitor of Rnr1. Although inactivation of Sml1 is required to maintain viability in *mec1Δ* and *rad53Δ* strains, Sml1 is intact in the partial loss-of-function *mec1-21* and *rad53-21* strains.

**Figure 4:**
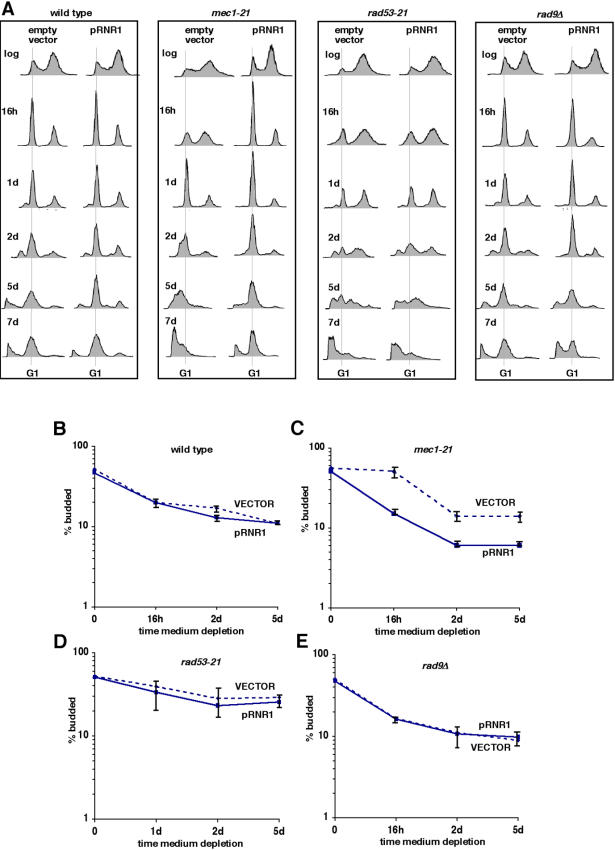
Ectopic expression of *RNR1* suppresses increased apoptotic DNA degradation and less efficient G1 arrest in *mec1-21*, but not *rad53-21* cells during nutrient depletion. A. DNA content of wild-type (W303) and indicated checkpoint mutant strains transformed with an empty vector or a high copy plasmid expressing *RNR1* (“pRNR1”) during nutrient depletion in SC medium. B–E. Budding index of these strains during nutrient depletion in SC medium. Error bars depict the standard error for measurements made in three or more independent experiments.

To determine whether failure to maintain dNTP pools might explain the effects of the *mec1-21* and *rad53-21* mutations, we analyzed the effects of ectopic expression of *RNR1* from a high copy plasmid. In some, but not all, experiments, ectopic expression of *RNR1* in wild-type or *rad9Δ* cells enhanced arrest with a G1 content of DNA induced by nutrient depletion (Fig. 4A, “wild type” and “*rad9Δ*”; Fig. 4B and E). This enhanced G1 arrest was accompanied by a reduction in the number of cells undergoing apoptotic DNA degradation at later time points (Fig. 4A “wild type”). This is consistent with the possibility that replication stress caused by insufficient dNTPs during nutrient depletion can trigger apoptosis. In most experiments, however, effects of ectopic expression of *RNR1* were not detected in wild-type or *rad9Δ* cells. We conclude that cells harboring wild-type Mec1 and Rad53 usually maintain sufficient levels of dNTPs at early stages of nutrient depletion to complete the replication of chromosomes before arresting in G1. The occasional effects of ectopic *RNR1* expression in wild type and *rad9Δ* cells may reflect a critical balance between the competing requirement during nutrient depletion to maintain sufficient dNTPs to complete S phase at early stages and to downregulate *RNR1* at later stages.

In contrast to the occasional effects of ectopic expression of *RNR1* in wild type and *rad9Δ* cells, ectopic expression of *RNR1* in *mec1-21* cells consistently suppressed the defective G1 arrest in these cells as measured by DNA content (Fig. 4A; “*mec1-21*”), as well as bud indices (Fig. 4C). It also suppressed increased apoptotic DNA degradation in these cells during nutrient depletion (Fig. 4A “*mec1-21*”). In fact, ectopic expression of *RNR1* reproducibly led to a more efficient G1 arrest at earlier time points in *mec1-21* compared to wild-type cells indicated by more cells with a G1 DNA content (Fig. 4A; compare “wild type pRNR1” with “*mec1-21* pRNR1”) and fewer buds (compare Fig. 4C “pRNR1” with Fig. 4B “pRNR1”). This may be due to the combined effects of elevated dNTP pools resulting from ectopic expression of *RNR1* and the premature activation of late S phase-firing replication origins that occurs in cells harboring mutations in *MEC1* [48], [49]. In the presence of excess dNTPs, activation of late replication origins in early-S-phase cells would accelerate exit from S phase during nutrient depletion. This may allow for exit from S phase before other factors required for DNA replication have been downregulated in response to nutrient depletion. In contrast to *mec1-21* cells, cells from which both *MEC1* and *SML1* had been deleted did not exhibit a defective G1 arrest or increased apoptosis (Fig. S3). This is consistent with a role for Mec1 in promoting G1 arrest during nutrient depletion related to its downregulation of Sml1 and increased ribonucleotide reductase activity. Downregulation of Sml1 would lead to the induction of ribonucleotide reductase activity and increased dNTP pools required for exit from S phase before other factors required for DNA replication have been downregulated.

In contrast to its effects in *mec1-21* cells, ectopic expression of *RNR1* had no detectable effect on the defective G1 arrest and increased apoptotic DNA degradation phenotypes of *rad53-21* cells (Fig. 4A; “*rad53-21*” and Fig. 4D). This may reflect the existence of lethal defects in Rad53-dependent, but Mec1-independent regulation of histone levels in cells subjected to DNA damage or replication stress [50]. An additional function of Rad53 that is not shared by Mec1 has been suggested by other studies as well [51], [52], [53], [54]. This includes the demonstration that *rad53Δ*, but not *mec1Δ* cells exhibit a slow-growth phenotype when Sml1 is inactivated in these cells [54], which suggests a Mec1-independent function of Rad53 that is not related to the regulation of nucleotide levels. We conclude that nutrient depletion-induced replication stress is mitigated in wild-type cells by Mec1-dependent induction of Rnr1 activity. Regulation of Rnr1 activity by Mec1 during nutrient depletion likely occurs in collaboration with Rad53, but in conjunction with other Mec1-and Sml1-independent effects of Rad53 that also impact nutrient depletion-induced G1 arrest.

### Ectopic expression of Cln3 abrogates G1 arrest, shortens lifespan and accelerates genome instability and apoptosis during nutrient depletion

The findings described above are consistent with the hypothesis that inhibition of nutrient signaling pathways extends chronological lifespan by promoting a more efficient arrest in G1 that protects against DNA replication stress. Progression past Start in G1 and entry into S phase require the activity of the Cdc28 cyclin-dependent kinase, which is regulated by the G1 cyclin Cln3 upstream of the cyclins Cln1 and Cln2 (reviewed in [55]). The expression of all three Clns is down-regulated in nutrient-depleted cells [37], [56]. We next asked whether ectopic expression of *CLN3* during nutrient depletion would attenuate the G1 arrest induced by nutrient depletion and shorten chronological lifespan in the absence of increased nutrient signaling.

In initial experiments, ectopic expression of *CLN3* paradoxically increased the fraction of viable cells at later time points (compared to empty vector-transformed control cells) in concert with increased apoptotic DNA degradation (Fig. S4). At later time points in chronological aging experiments, strains with shorter chronological lifespans often exhibit a phenomenon called adaptive regrowth, which occurs stochastically in association with the release of substances from apoptosing cells into the medium. These substances promote the growth of cells that at earlier time points were growth-arrested due to nutrient depletion [14], [15]. Adaptive regrowth could explain the relative increase in viable cells detected at later time points in populations of cells ectopically expressing *CLN3*, despite an increased frequency of apoptosis in these cell populations (Fig. S4). Consequently, in subsequent experiments, we measured the effects of ectopic *CLN3* expression on chronological lifespan under conditions that avoided adaptive regrowth by periodically reseeding cells in medium that had been pre-depleted of nutrients. This blocked the accumulation of substances released from apoptosing cells without altering the nutrient-depleted status of surviving cells.

Pre-depleted medium was prepared by long-term (seven days) culture of *sch9Δ* cells, since these cells are less susceptible (compared to wild type cells) to apoptosis (Fig. 1A) or adaptive regrowth [29]. *CLN3*-overexpressing and vector-control cells were reseeded into this pre-depleted medium (from which *sch9Δ* cells had been removed) every other day beginning one day after cultures of these cells were first established. Analysis of wild type and *sch9Δ* cells cultured for an extended time period using this regimen indicated that periodic reseeding in pre-depleted medium did not alter the chronological lifespan of wild type cells or the tighter G1 arrest and reduced apoptotic DNA degradation that occurs in *sch9Δ* cells (Fig. S5). Therefore, the use of pre-depleted medium does not alter the mechanisms by which nutrient signaling pathways impact chronological lifespan.

**Figure 5:**
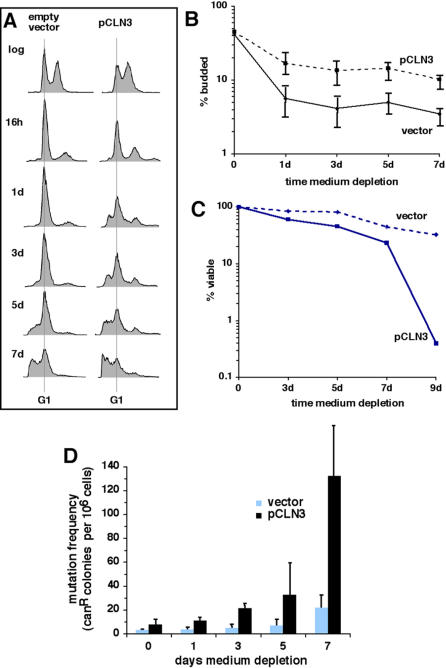
Ectopic expression of *CLN3* during nutrient depletion shortens lifespan and accelerates age-dependent genome instability and apoptosis. A–C. DNA content (A), budding index (B) and viability (C) during nutrient depletion of cells transformed with an empty vector or a plasmid expressing *CLN3* using the *CUP1* promoter (“pCLN3”). D. Mutation frequency during nutrient depletion measured by resistance to canavanine in cells transformed with an empty vector or with pCLN3. Error bars depict the standard error for measurements made in three independent experiments. An exception is canavanine resistance measurements in cells transformed with pCLN3 at day 5 (panel D), where it represents the range of values from two of three independent experiments (due to a technical problem at this timepoint in one experiment).

In pre-depleted medium, cells ectopically expressing *CLN3* reproducibly exhibited a less efficient G1 arrest compared to cells transformed with an empty vector, and frequently arrested growth in S phase instead (Fig. 5A and B). The less efficient growth arrest in G1 of cells ectopically expressing *CLN3* was accompanied by accelerated apoptotic DNA degradation (Fig. 5A) and a shorter chronological lifespan (Fig. 5C; note logarithmic scale). Shorter chronological lifespan associated with ectopic expression of *CLN3* during nutrient depletion was recently reported by others as well [57]. The shorter lifespan of *CLN3*-expressing cells during nutrient depletion is similar to reports of shortened lifespan associated with ectopic expression of *CLN3* in cells undergoing growth arrest induced by inhibiting TOR signaling pathways with rapamycin [57], [58].

Genome instability is an important component of chronological aging in budding yeast [29] and other organisms [8]. To determine whether the less efficient growth arrest in G1 and shortened lifespan of nutrient-depleted cells ectopically expressing *CLN3* are accompanied by accelerated age-dependent genome instability, we asked whether these cells suffer an increase in the frequency of mutations in the *CAN1* gene, as measured by increased resistance to the toxic amino-acid analogue canavanine. We found that, similar to a previous report [29], nutrient depletion induced a chronological-age-dependent increase in mutation frequency in wild-type cells (Fig. 5D; “vector”). This mutation frequency was dramatically elevated in chronologically aged cells ectopically expressing *CLN3* (Fig. 5D; “pCLN3”). Therefore, in addition to shortening chronological lifespan and stimulating apoptosis, the failure to efficiently arrest growth in G1 during nutrient depletion contributes to chronological age-dependent genome instability.

## Discussion

### Impact of altered nutrient signaling on chronological lifespan

**Figure 6:**
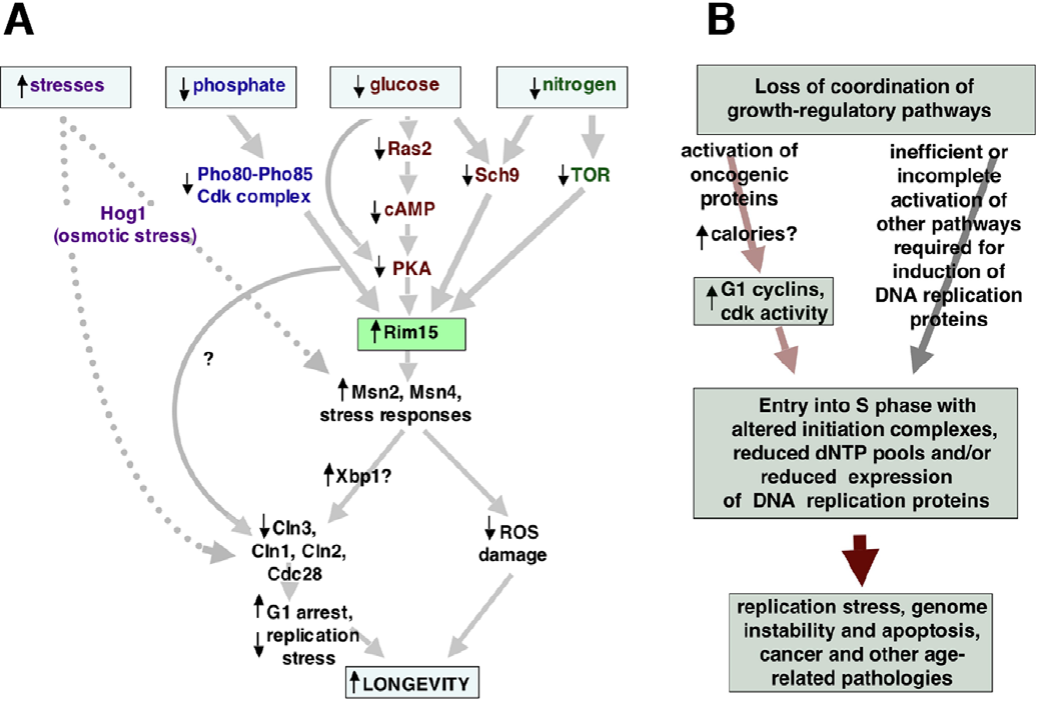
Models for longevity regulation by growth signaling pathways in budding yeast (A) and deregulation of growth regulatory pathways leading to replication stress and aging in all eukaryotes (B). A. In budding yeast, nutrient signaling pathways that respond to glucose, nitrogen and phosphate converge upon Rim15, which is downregulated by signaling through these pathways when nutrients are plentiful. Activation of Rim15 when nutrient signaling is inhibited induces stress response factors (including Msn2 and Msn4) and stress responses mediated by Sod1, Sod2 and other proteins that mitigate oxidative damage and other effects of stresses. Reduced nutrient signaling also downregulates Cln3 and downstream Clns1 and 2, which are required for activation of the cyclin-dependent kinase Cdc28. Inactivation of Cdc28 during nutrient depletion contributes to a G1 arrest that protects against replication stress. Osmotic stress (and perhaps other stresses) also contribute to G1 arrest during nutrient depletion. G1 arrest in response to nutrient depletion and other stresses may be mediated in part by induction of the transcriptional repressor Xbp1, which inhibits Cln transcription. Enhancement of G1 arrest by caloric restriction, osmotic stress or mutational inactivation of nutrient signaling pathways protects against replication stress and promotes longevity in combination with Rim15-dependent responses to oxidative-and other stresses. B. Replication stress associated with uncoordinated entry into or exit from S phase downstream of the activation of some, but not all, growth signaling pathways may be an important factor determining lifespan in all eukaryotes. In both panels, question marks indicate hypothetical or undefined effects.

Most efforts to understand how alterations in nutrient signaling pathways impact the chronological lifespan of budding yeast have focused on changes in oxidative stress responses regulated by these pathways downstream of Rim15. Our findings point to an efficient Rim15-dependent growth arrest in G1 that also requires downregulation of Cln3 as an additional factor determining chronological lifespan in this organism (Fig. 6A). Caloric restriction, mutational inactivation of Sch9 or Ras2 and growth in YPD rather than SC medium enhance this G1 arrest and extend lifespan. In contrast, constitutive activation of nutrient signaling by *RAS2* ^val19^ or deletion of *RIM15* increases the frequency with which nutrient-depleted cells growth-arrest in S phase instead of G1 and shortens chronological lifespan. During nutrient depletion, sustained expression of *CLN3*–which is normally downregulated under these conditions–also increases the frequency with which cells growth arrest in S phase in concert with a shorter lifespan and age-dependent increases in genome instability and apoptosis. Therefore, downregulation of Cln3 is required for G1 arrest and long-term survival in nutrient-depleted cells. Importantly, the effects of ectopic *CLN3* expression occur in the absence of altered nutrient signaling that would impact oxidative stress. Therefore, changes in the efficiency of G1 arrest associated with altered nutrient signaling can impact chronological lifespan and age-dependent genome stability independently of changes in stress responses that would affect oxidative damage to DNA and other cellular constituents.

How downregulation of Cln3 occurs during nutrient depletion remains unclear. Xbp1, a transcriptional repressor of *CLN* genes, may contribute to this downregulation. *XBP1* is induced by nutrient depletion, and ectopic expression of Xbp1 in cycling cells slows growth by lengthening G1 phase [59]. Induction of *XBP1* by nutrient depletion may occur downstream of Rim15 activation and its induction of Msn2 and Msn4. The promoter for *XBP1* harbors binding sites for these and other stress factors induced by Rim15 activation during nutrient depletion [59]. Msn2 and Msn4 antagonize PKA-dependent growth [60]. This establishes a role for these stress response factors in growth regulation–perhaps upstream of the induction of *XBP1*-in addition to the role they play in responses to oxidative and other stresses.

Nutrient depletion-induced G1 arrest is enhanced by osmotic stress, similar to the effects on G1 arrest of mutational inactivation of nutrient signaling pathways and caloric restriction (Fig. 2). In cycling cells, osmotic stress inhibits growth by inducing a G1 arrest accompanied by the downregulation of Clns and Cdc28 activity. This G1 arrest depends on stabilization of the Cdc28 inhibitor Sic1 by the Hog1 MAP kinase [61]. Hog1 may also downregulate the expression of Clns in osmotically stressed cells, which would contribute to Sic1 stability [61]. The *CLN* transcriptional repressor *XBP1* is also induced by osmotic stress [59], and therefore may contribute to the downregulation of Clns and Cdc28 activity in osmotically stressed cells. Similar to ectopic expression of *CLN3* during medium depletion, abrogation of the Hog1-dependent G1 arrest in osmotically stressed cells is accompanied by genome instability [61]. We think it is likely that the chronological lifespan-extending effects of osmotic stress during nutrient depletion are related to enhanced downregulation of Clns and Cdc28 activity by Hog1-and Xbp1-dependent mechanisms that leads to a tighter nutrient depletion-induced G1 arrest.

### Replication stress and chronological lifespan

What is the mechanism by which a more efficient G1 arrest during nutrient depletion enhances chronological lifespan? Our results also indicate that nutrient depletion causes DNA replication stress. The induction of replication stress during nutrient depletion is indicated by the finding that cellular responses mediated by Mec1 and Rad53 (which respond to replication stress and DNA damage) are more important to the survival of nutrient-depleted cells than responses mediated by Rad9 (which responds to DNA damage, but not replication stress). At least at initial stages of nutrient depletion, Mec1 mitigates replication stress, most likely by increasing levels of dNTPs (Figs. 3 and 4). This is indicated by the ability of ectopically expressed *RNR1* to suppress the shorter lifespan and increased apoptosis in nutrient-depleted *mec1-21* cells (Fig. 4). Ectopic expression of *RNR1* would have the effect of increasing dNTP pools that in wild type (but not *mec1-21*) cells are upregulated by Mec1 and Rad53 in response to replication stress. Mec1 and Rad53 upregulate ribonucleotide reductase by a mechanism that leads to destabilization of the ribonucleotide reductase inhibitor Sml1. Therefore, replication stress at early stages of nutrient depletion is mostly or entirely a consequence of starvation for dNTPs. Consistent with this possibility, *mec1Δ* cells from which *SML1* has also been deleted (which leads to increased levels of dNTPs [54]) are phenotypically similar to wild type cells (Fig. S3). The absence of effects in *mec1Δ sml1Δ* compared to wild type cells also suggests that the checkpoint function of Mec1, which is separate from its role in regulating dNTP metabolism, does not play a role in regulating chronological lifespan.

Deletion of *RAD9* also shortens lifespan and accelerates apoptosis, although not to the same extent as the *mec1-21* and *rad53-21* mutations (Fig. 3). Therefore, nutrient depletion likely induces DNA damage in addition to replication stress. Nutrient depletion-induced DNA damage is consistent with the age-dependent accumulation of mutations during chronological aging (Fig. 5D and [29]). Importantly, the induction of DNA damage or replication stress during nutrient depletion is not inconsistent with our failure to detect a role for Mec1-dependent checkpoint responses in the survival of nutrient-depleted cells. Mec1-dependent pathways are inactivated by superoxide anions [62], which accumulate during nutrient depletion [63]. Since Mec1-dependent pathways also contribute to the stability of stalled replication forks [64], inactivation of these pathways by superoxide anions at later stages of nutrient depletion might contribute to DNA damage in nutrient-depleted cells undergoing replication stress.

Although replication stress-induced recombination in the rDNA locus is a major determinant of budding yeast replicative lifespan ([13] and references therein), replication stress has not been considered a factor in chronological aging in this organism. Most likely this is because during nutrient depletion, most cells appear to arrest without buds and with a G1 content of DNA, which suggests they are not in S phase. It is difficult to detect minor increases in DNA content by flow cytometry, however, and small buds are difficult to detect microscopically. Furthermore, the activation of some, but not all, nutrient signaling pathways may lead to uncoupling of events required for budding from progression past Start. Consequently, although DNA content and bud counts provide useful relative measures of the number of cells that arrest in G1 during nutrient depletion, they likely overestimate the number of G1 cells and underestimate the number of cells that arrest growth while in S phase.

This view is consistent with a recent report that nutrient depletion leads to the accumulation of a substantial fraction of less dense cells, which includes all the cells that remain budded during nutrient depletion plus many cells that do not appear to have buds [65]. These less-dense cells differentially express a number of genes encoding proteins required for the resolution of stalled DNA replication forks, suggesting they are under replication stress. These cells also more frequently undergo apoptosis compared to denser cells that do not express these genes. These phenotypes are consistent with our proposed role for nutrient depletion-induced replication stress in chronological aging.

### How does nutrient depletion cause replication stress in S phase cells?

Several factors likely contribute to replication stress in cells that arrest growth in S phase during nutrient depletion. In addition to the potential depletion from medium of substrates required for synthesis of dNTPs, these factors include downregulation of genes encoding proteins required for DNA replication [44], [45], [46]. Interestingly, ectopic expression of the constitutively activating *RAS2* ^val19^ mutation induces transcription of *CLN3*, but not transcription of *RNR1* and other DNA replication-related genes ([66], including supplementary information). Consequently, during nutrient depletion, cells harboring the *RAS2* ^val19^ mutation may enter S phase with a reduced capacity to replicate DNA. Similar uncoordinated entry into S phase when only a subset of growth-regulatory pathways are active could explain an earlier report of growth-promoting, but lethal apoptosis-inducing effects of glucose added to stationary phase cultures in the absence of other nutrients [67].

Aberrant initiation of DNA replication is another factor that likely contributes to replication stress in nutrient-depleted cells. Deregulated Cln expression inhibits the assembly of pre-replicative complexes (pre-RCs) required for initiation of DNA replication at replication origins [68]. Inhibition of pre-RC assembly induces genome instability [68], [69], as well as DNA damage [69] and apoptosis [28].

### Caloric restriction and chronological lifespan

In cycling budding yeast cells, the effects of caloric restriction on replicative lifespan are related to reduced signaling through PKA, TOR and Sch9-dependent pathways, because caloric restriction does not extend replicative lifespan further when these pathways have been inactivated [70], [71]. Our findings are consistent with the possibility that caloric restriction during nutrient depletion extends chronological lifespan via a similar reduction in signaling through these pathways, which leads to a tighter nutrient depletion-induced G1 arrest and reduced replication stress (Fig. 2). This tighter G1 arrest may be related to the accelerated downregulation of *CLN3* mRNA when glucose concentration is reduced [37].

Both caloric restriction and deletion of *CLN3* suppress the shorter chronological lifespan of nutrient-depleted polyploid compared to haploid cells in parallel with a more efficient G1 arrest [72]. This is also consistent with the possibility that caloric restriction can extend chronological lifespan by downregulating Cln3. Although deletion of *CLN3* extended the chronological lifespan of polyploid cells, it did not extend the chronological lifespan of haploid cells in this prior study or in our experiments (not shown). This might reflect the existence of redundant, Cln3-independent pathways that contribute to G1 arrest during nutrient depletion, some of which may be inoperative in polyploid cells. Whatever the explanation, our experiments clearly suggest that the increased chronological lifespan of *cln3Δ* or calorie-restricted polyploid cells reported in this earlier study was related to attenuation of replication stress.

### Replication stress and hormesis effects on aging

Many of the genes induced by nutrient depletion are also induced by other stresses, including oxidative and osmotic stress, heat and DNA damage [45]. Specific stresses often confer cross-resistance to other stresses, for reasons that are not clear. Cross-resistance to multiple stresses and the lifespan-extending effects associated with mild exposures to these stresses is the basis for the “hormesis hypothesis” of aging [33]. This hypothesis posits a general stress response induced by caloric restriction and other stresses that protects against a variety of different stresses as an important component of longevity in all eukaryotes. The nature of this putative general stress response is not clear.

In addition to nutrient depletion and osmotic stress, other stresses including oxidative stress [73], heat [74], and DNA damage [75]—all of which also have been implicated in hormesis effects that extend lifespan—inhibit growth by inducing a G1 arrest in budding yeast. The *CLN* transcriptional repressor Xbp1 is induced by all these stresses, in addition to its induction by nutrient depletion and osmotic stress [59]. The growth-inhibitory effects of stresses and the hormesis-like lifespan-extending effects of osmotic stress in concert with a tighter G1 arrest reported here (Fig. 2) clearly point to the existence of a general response to environmental stresses that enhances longevity by inhibiting growth, and thus replication stress and age-dependent genome instability. Inhibition of replication stress under these conditions likely contributes to cross-resistance to various stresses that underlies the hormesis hypothesis. Similar to genes induced by nutrient depletion, genes induced by other stresses are nonrandomly distributed in the budding yeast genome and are more likely to be repressed by chromatin structure in the absence of stress. This is consistent with the possibility that hormesis effects of stresses on lifespan in budding yeast are coordinately regulated by chromatin structure within clusters of stress-related genes [76].

### Replication stress and aging in higher eukaryotes

In addition to their effects on replicative and chronological lifespan in budding yeast, caloric restriction, low levels of stress and mutations that inactivate growth signaling pathways extend the lifespans of many higher eukaryotes as well [1]. We propose that, similar to budding yeast, these factors promote longevity in all eukaryotes by inhibiting replication stress associated with uncoordinated entry into or exit from S phase (Fig. 6B). This may be particularly important during differentiation leading to a quiescent, non-dividing state, which requires downregulation of the Cln3 homologue cyclin D1. Consistent with this model, in mice, caloric restriction extends lifespan in concert with a reduction in the number of cells in S phase in a variety of tissues containing differentiating cells, including intestinal epithelium, spleen, thymus and mesenteric lymph nodes [77]. Other studies have also detected a relationship between caloric restriction and reduced cellular proliferation in higher eukaryotes (reviewed in [78]).

In fact, accumulating evidence points to replication stress downstream of aberrant growth signaling as a determinant of lifespan in higher eukaryotes, including humans. This includes the recent discovery that replication stress-induced DNA damage and apoptosis are present in preneoplastic human cells that eventually give rise to various cancers, for which age is a dominant risk factor [79], [80]. Replication stress-induced DNA damage and apoptosis at early stages of neoplasia likely arise downstream of the mutational activation of genes that promote growth, similar to the constitutive activation of *RAS2* by the *RAS2* ^val19^ mutation in budding yeast. For example, it was recently shown that sustained mitogenic signaling induced by ectopic expression of activated *RAS* in quiescent rat fibroblasts stimulates G1 cyclin-dependent kinase activity, entry into S phase, genome instability and apoptosis [81], [82], all of which correspond to phenotypes detected in the yeast experiments reported here. Similar to the *RAS2* ^val19^ mutation, which induces *CLN3* but not genes encoding proteins required for efficient DNA replication [66], the oncogenic activation of Ras and other proteins in mammalian cells may induce a subset of the complex, interacting growth-regulatory pathways normally required for progression into and through S phase. Induction of a subset of these pathways in the absence of the parallel induction of proteins required for efficient DNA replication would create replication stress (Fig. 6B). Depending on the type of cell and the status of cell cycle checkpoint pathways, DNA damage produced by replication stress could induce a number of aging phenotypes. In addition to neoplastic transformation, these include senescence of stem cells or their proliferating progeny and cell death.

Age is also a dominant risk factor for many neurodegenerative diseases [83]. Apoptosis associated with entry into S phase and inefficient DNA replication in normally quiescent postmitotic neurons has been implicated in the etiology of a number of these diseases as well ([84] and references therein). In fact, a causal role for the activation of TOR signaling pathways in neurodegeneration was recently demonstrated in a *Drosophila* model of neurodegenerative disease [84]. Although this role can be explained by other models [84], our findings suggest replication stress as a potential explanation.

In summary, the findings reported here point to DNA replication stress downstream of deregulated nutrient signaling as an important determinant of chronological aging in budding yeast. They suggest that caloric restriction and other experimental manipulations that inhibit growth—including mild stresses—can extend chronological lifespan by decreasing the frequency with which cells arrest growth in S phase during nutrient depletion. This reduces replication stress, as well as age-dependent DNA damage and genome instability caused by replication stress. Accumulating evidence suggests that replication stress induced by aberrant growth regulation is an important factor in age-related pathologies in higher eukaryotes, including humans. Although the destabilizing effect of replication stress in the rDNA locus associated with replicative aging in budding yeast has not been detected in other eukaryotes, a universal role for replication stress in aging is suggested by the fact that that in many eukaryotic organisms, mutations in RecQ helicases required for accurate and efficient DNA replication promote genome instability and premature aging (reviewed in [7], [85], [86]. Our findings suggest that some of the genome instability associated with aging is related to DNA damage caused by replication stress instead of oxidative stress. Deregulated growth signaling and inappropriate entry into, or exit from, the cell cycle in nutrient-starved budding yeast cells provide a new paradigm for investigating how oncogenic activation of growth-regulatory pathways and the induction of replication stress in mammalian cells contribute to cancer and other age-related diseases.

## Materials and Methods

### Yeast strains and plasmids

Strain backgrounds employed in this study were DBY746 (MATa leu2-3, 112, his3Δ1 trp1-289, ura3-52, GAL^+^); W303-1A (MATa, ade2-1, ura3-1, his3-11, trp1-1, leu2-3, can1-100), JC482 (MATα, leu2, ura3, his4) and BY4741 (MATa, his3Δ1, leu2Δ0, met15Δ0, ura3Δ0). Mutant strains employed were PF102 (DBY746 sch9::URA3); MWY420 (DBY746 rim15::TRP1); rim15Δ sch9Δ (DBY746 sch9::URA3 rim15::LEU2); RAS2^val19^ (JC482 RAS2^val19^); Y604 (W303 mec1-21); Y301 (W303 rad53-21); HKY845 (W303 rad9::HIS3). All strains in the DBY746 background were from V. Longo (USC) except MWY420, which was constructed for this study. Strains in the JC482 background were from M. Breitenbach (University of Salzburg). Y604 and Y301 were from S. Elledge (Harvard). HKY845 was from H. Klein (NYU). The plasmid Yep-RNR1 was from E. Vallen, Swarthmore College. Yep 24 (vector control for YEp24-RNR1) was purchased from ATCC. pCCul expressing CLN3 from the CUP1 promoter and its control vector pJ16 were from W. Heideman (U. of Wisconsin). pSR17 containing rim15::TRP employed for construction of MWY420 and PMF100 containing ras2^val19^ (employed in the construction of MWY421) were from V. Longo (USC). To construct MWY420, rim15Δ::TRP1 was excised as a Xho1-Sal1 fragment from pSR117 and inserted in DBY746 by transformation and selection for TRP^+^ growth. To construct MWY421, RAS2^val19^ :: URA3 was excised as an EcoR1-HindIII fragment from pMF100 and inserted into W303 by transformation and selection for URA^+^ growth.

### Cell culture conditions, flow cytometry measurements of DNA content, bud measurements

To assess chronological lifespan, cells from exponentially proliferating cultures were inoculated into 50 mls. SC or YPD medium containing 2% glucose [87] in 250 ml. flasks at an initial density of 5×10^5^/ml. and continuously cultured at 30°C with rotary shaking for indicated times. To assess the effects of osmotic stress, sorbitol was added to these cultures to a final concentration of 1M. For experiments requiring ectopic expression of *CLN3*, SC medium was pre-depleted by growth of *sch9Δ* cells for 7 days followed by pelleting of cells and filter sterilization of medium. Exponential cultures of DBY746 cells transformed with pCLN3 or a vector control plasmid were seeded into standard SC medium. Beginning 24 hours later, cells were pelleted from medium and resuspended in pre-depleted medium every other day throughout the course of experiments.

To assess the effects of caloric restriction on chronological lifespan, an equal number of cells from exponentially proliferating cultures were pelleted by centrifugation, washed twice with fresh medium containing 0.5% glucose and then seeded into this latter medium or into fresh medium containing 2% glucose. Caloric restriction experiments employed a slightly different formulation of SC medium [88] compared to the formulation employed in other experiments [87]. However, repetition of these experiments in the SC formulation employed in other experiments confirmed that the effects of caloric restriction were the same in both formulations (not shown).

In all chronological lifespan measurements, aliquots removed from cultures at the indicated times were plated in triplicate on YPD agar to determine survival (as colony forming units). To determine budding indices and DNA content, cells in aliquots taken at each time point were pelleted by centrifugation and resuspended in water (for determining budding index) or 70% ethanol (for flow cytometry). The budding status of at least 500 cells from each aliquot was visually determined using a Nikon Eclipse E600 microscope with a 40× phase contrast objective. Just before examining cells, cell clumps were dissociated by sonication using a Model 60 Sonic Dismembrator sonicator (Fisher Scientific, Hampton, NH) for 10 seconds at power setting 5. To measure DNA content by flow cytometry, cells suspended in 70% ethanol were pelleted by centrifugation, washed with 50 mM sodium citrate (pH 7.5) and resuspended in 0.5 ml of this same buffer containing 0.5 mg/ml RNAse. After overnight incubation at 37°C, an additional 0.5 ml. of sodium citrate buffer containing 2 µM SYTOX Green (Invitrogen, Carlsbad, CA) was added to each sample. Stained cells were briefly sonicated as described above and DNA content was measured using a FacsCaliber flow cytometer (BD Biosciences, Woburn, MA) at a maximum flow rate of 500 cells/s. Flow cytometry data were processed using CellQuest (BD Biosciences) and Flojo (Tree Star Inc., Ashland, OR) software. Y axis scales indicating number of cells were maintained constant in all flow cytometry profiles for individual experiments.

### Measurements of mutation frequency

Mutation frequency was assessed by determining frequency of resistance to the toxic amino-acid analogue canavanine conferred by mutations in the *CAN1* gene. An appropriate number of cells were plated on selective medium containing 60 mg/l L-canavanine instead of arginine and canavanine-resistant colonies were counted 3–5 days later.

### Cell Volume Measurements

Analysis of cell volume was performed using the Z2 Coulter Particle Count and Size Analyzer. DBY746 and BY4741 strains were grown for 2 days at 30°C in 5 ml of either SC+2% glucose or SC+0.5% glucose to saturation (performed in triplicate for each condition). Cells from these cultures were inoculated into fresh media to an OD_600_ of 0.1 in 10 ml of the same media. 1.0 OD of cells was collected at 6 hours, 24 hours, 72 hours, and 168 hours. The cells were spun down and resuspended in 100 µl sterile ddH_2_O and immediately analyzed. The 100 µl volume was added to 10 ml of azide-free H_2_O (Fisher Scientific) and analyzed on the Coulter counter (dilution factor = 100; range: 10 to 250 fL (femto-liters)). Prior to measurements, the cells were sonicated for ∼10s to break up any aggregates (duty cycle = 30%; continuous pulse). The mean cell volume in fL was manually calculated from the peak distribution.

### Measurements of mitochondrial DNA

Cells were isolated at indicated times and after fixation in 70% ethanol, DNA was stained with DAPI as described previously [89] to detect nuclear and cytoplasmic DNA. Photomicrographs of stained cells were obtained at 400× magnification with a Zeiss Axioskop microscope equipped with an Optronics Magnafire CCD camera. To determine relative amounts of mitochondrial compared to nuclear DNA, total yeast DNA was digested with the restriction enzyme XmnI, which produces a 6.3 kb fragment of nuclear DNA containing the *ACT1* gene and a 10.2 kb fragment of mitochondrial DNA containing sequences encoding 15S ribosomal RNA. XmnI-digested DNA was separated on 1% agarose gels and transferred to Duralon hybridization membranes. Membranes were probed with ^32^Phosphate-labeled DNA produced by PCR amplification of sequences within each of these DNA fragments. Radioactive signals from membranes were captured using a Storm PhosphoImager (GE Healthcare).

## Supporting Information

Figure S1 Chronological lifespan of sch9D compared to wild-type cells. Weinberger et al.
(0.05 MB PDF)

Figure S2 Effect of osmotic stress.
(0.13 MB PDF)

Figure S3 In the absence of the ribonucleotide reductase inhibitor Sml1, deletion of MEC1 does not alter the efficiency of G1 arrest during nutrient depletion. A. FACS measurements of DNA content in wild type and mec1D smlD cells at various times of nutrient depletion (indicated at left of FACS profiles). B. Budding index of these cells.
(0.18 MB PDF)

Figure S4 Ectopic expression of CLN3 paradoxically increases viability compared to vector-transformed control cells at the same time that it increases apoptotic degradation of DNA. A. Viability of vector-transformed cells (“VECTOR”) and cells ectopically expressing CLN3 (“pCLN”) during nutrient depletion. B. DNA content of these cells. Note that after 5 days of nutrient depletion, more cells ectopically expressing CLN3 are viable compared to vector-transformed cells (panel A), but a larger fraction of these cells exhibit less than a G1 content of DNA.
(0.17 MB PDF)

Figure S5 sch9D induces a tighter G1 arrest and suppresses apoptosis in cells cultured in pre-depleted medium, similar to the effects of culturing these cells in medium that was not pre-depleted (compare to Fig. 1A). Weinberger et al.
(0.29 MB PDF)
