# Supplementary material for: DNA Replication Stress Is a Determinant of Chronological Lifespan in Budding Yeast
Source: PLoS One. 2007 Aug 15;2(8):e748. doi: 10.1371/journal.pone.0000748 (PMC1939877; doi:10.1371/journal.pone.0000748)
Supplement: Figure S1 — Chronological lifespan of sch9D compared to wild-type cells. Weinberger et al. (0.05 MB PDF) [file pone.0000748.s001.pdf]

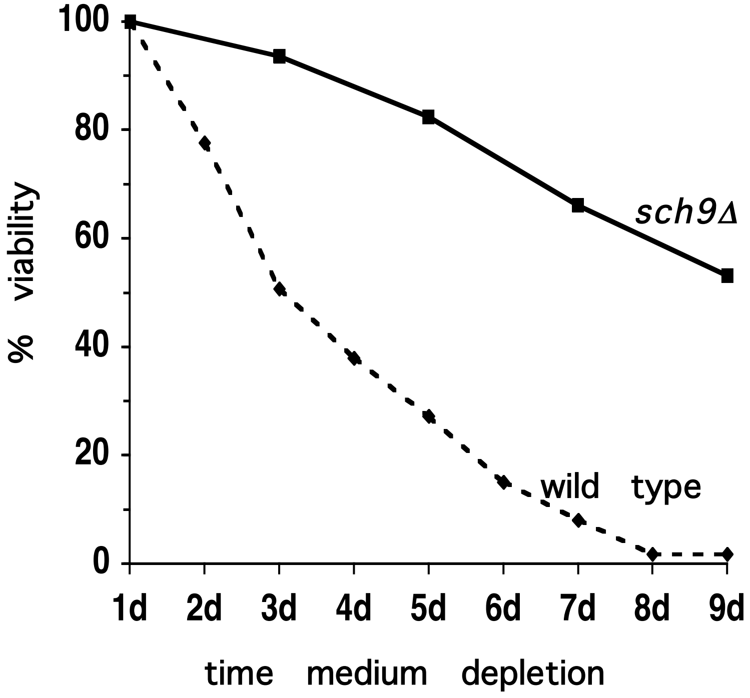

Fig. S1 Weinberger et al. Chronological lifespan of *sch9Δ* compared to wild-type cells (DBY746 background)
