# Supplementary figures and images for: DNA Replication Stress Is a Determinant of Chronological Lifespan in Budding Yeast
Source: PLoS One. 2007 Aug 15;2(8):e748. doi: 10.1371/journal.pone.0000748 (PMC1939877; doi:10.1371/journal.pone.0000748)

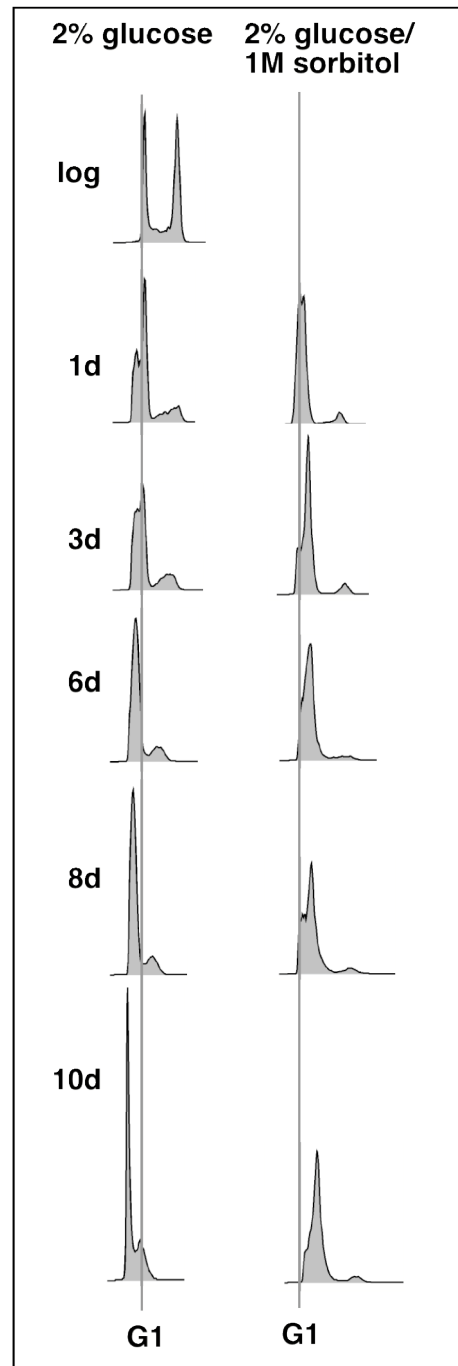

**Weinberger Fig. S2.** Effect of osmotic stress (1M sorbitol) on DNA content during medium depletion.

Supplement: Figure S2 — Effect of osmotic stress. (0.13 MB PDF) [file pone.0000748.s002.pdf]
