# Supplementary material for: DNA Replication Stress Is a Determinant of Chronological Lifespan in Budding Yeast
Source: PLoS One. 2007 Aug 15;2(8):e748. doi: 10.1371/journal.pone.0000748 (PMC1939877; doi:10.1371/journal.pone.0000748)
Supplement: Figure S3 — In the absence of the ribonucleotide reductase inhibitor Sml1, deletion of MEC1 does not alter the efficiency of G1 arrest during nutrient depletion. A. FACS measurements of DNA content in wild type and mec1D smlD cells at various times of nutrient depletion (indicated at left of FACS profiles). B. Budding index of these cells. (0.18 MB PDF) [file pone.0000748.s003.pdf]

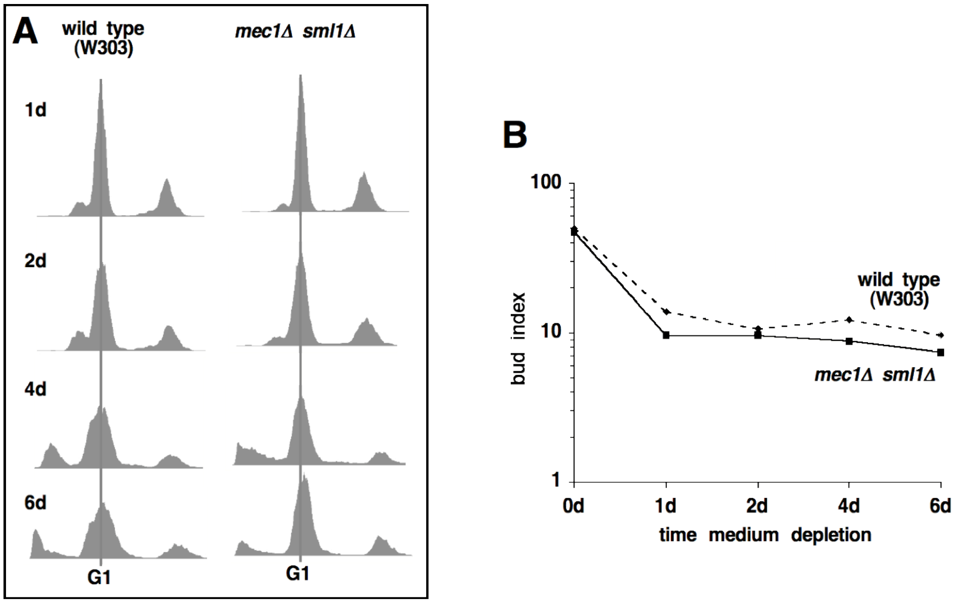

Figure S3 Weinberger et al. In the absence of the ribonucleotide reductase inhibitor Sml1, deletion of *MEC1* does not alter the efficiency of G1 arrest during nutrient depletion. **A.** FACS measurements of DNA content in wild type and *mec1Δ sml1Δ* cells at various times of nutrient depletion (indicated at left of FACS profiles). **B.** Budding indices of these cells.
