# Supplementary material for: DNA Replication Stress Is a Determinant of Chronological Lifespan in Budding Yeast
Source: PLoS One. 2007 Aug 15;2(8):e748. doi: 10.1371/journal.pone.0000748 (PMC1939877; doi:10.1371/journal.pone.0000748)
Supplement: Figure S4 — Ectopic expression of CLN3 paradoxically increases viability compared to vector-transformed control cells at the same time that it increases apoptotic degradation of DNA. A. Viability of vector-transformed cells (“VECTOR”) and cells ectopically expressing CLN3 (“pCLN”) during nutrient depletion. B. DNA content of these cells. Note that after 5 days of nutrient depletion, more cells ectopically expressing CLN3 are viable compared to vector-transformed cells (panel A), but a larger fraction of these cells exhibit less than a G1 content of DNA. (0.17 MB PDF) [file pone.0000748.s004.pdf]

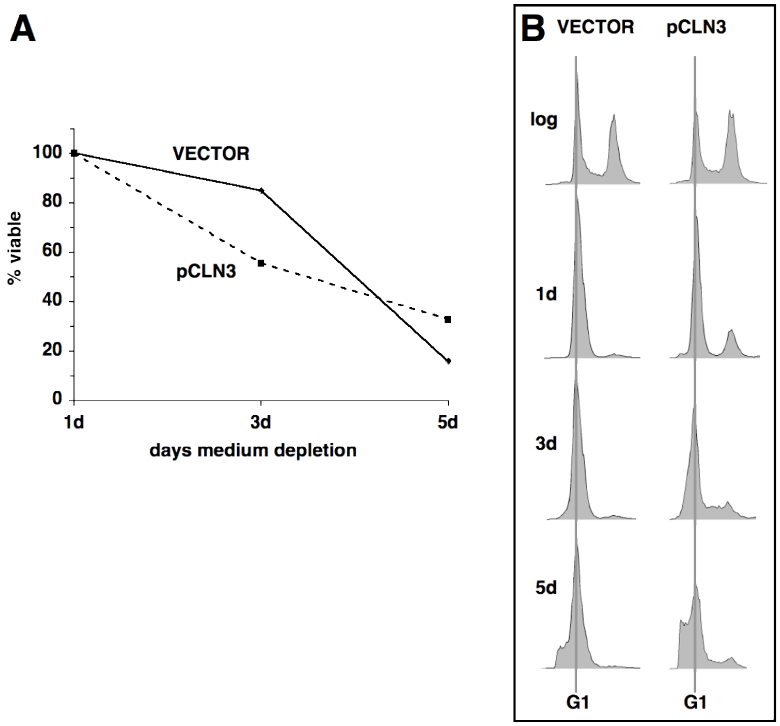

Figure S4. Ectopic expression of *CLN3* paradoxically increases viability compared to vector-transformed control cells at the same time that it increases apoptotic degradation of DNA. A. Viability of vector-transformed control cells "VECTOR" and cells ectopically expressing *CLN3* ("pCLN3") during nutrient depletion. **B.** DNA content of these cells. Note that after 5 days of nutrient depletion, more cells ectopically expressing *CLN3* are viable compared to vector-transformed cells (Panel A) but a larger fraction of these cells also exhibit less than a G1 content of DNA, indicating apoptotic DNA degradation (Panel B).
