# Supplementary material for: DNA Replication Stress Is a Determinant of Chronological Lifespan in Budding Yeast
Source: PLoS One. 2007 Aug 15;2(8):e748. doi: 10.1371/journal.pone.0000748 (PMC1939877; doi:10.1371/journal.pone.0000748)
Supplement: Figure S5 — sch9D induces a tighter G1 arrest and suppresses apoptosis in cells cultured in pre-depleted medium, similar to the effects of culturing these cells in medium that was not pre-depleted (compare to Fig. 1A). Weinberger et al. (0.29 MB PDF) [file pone.0000748.s005.pdf]

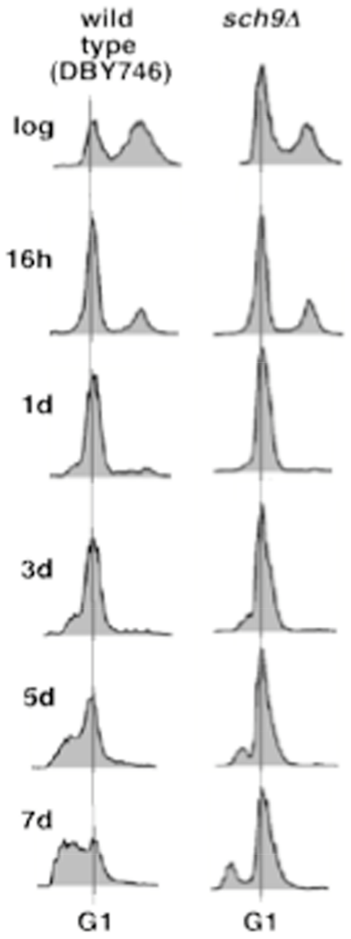

Fig. S5. *sch9Δ* induces a tighter G1 arrest and suppresses apoptosis in cells cultured in pre-depleted medium, similar to the effects of culturing these cells in medium that was not pre-depleted (compare to Fig. 1A)
